# MID-LIFE PARENTS' MATERIAL SUPPORT OF CHILDREN IN COLLEGE: A TYPOLOGY AND VARIATION BY SOCIOECONOMIC STATUS

**DOI:** 10.1093/geroni/igac059.225

**Published:** 2022-12-20

**Authors:** Tanya Whitworth

**Affiliations:** Boston University, Boston, Massachusetts, United States

## Abstract

Many imagine the “typical” college student to be 18-22 years old, attending a four-year institution, living on campus, and receiving financial support from parents. This perception does not align with actual patterns. While some parents facilitate this “traditional college experience” for their children, others substitute co-residence for or combine co-residence with financial support; still others provide no material support at all. Using a diverse sample of U.S. college students from the Panel Study of Income Dynamics Transition into Adulthood Supplement (n = 1,579), this paper demonstrates variation in material support provided by parents. I employ a latent class approach to categorize students into assistance “types,” and then use multilevel multinomial logistic regression models to predict assistance types from parents’ socioeconomic status (SES). Lower SES parents have a higher probability of providing co-residence, while higher SES parents have a higher probability of providing financial support to students living away from home.

